# Exercise training improves heart rate recovery in women with breast cancer

**DOI:** 10.1186/s40064-015-1179-0

**Published:** 2015-08-01

**Authors:** Francesco Giallauria, Luigi Maresca, Alessandra Vitelli, Maria Santucci de Magistris, Paolo Chiodini, Amalia Mattiello, Marco Gentile, Maria Mancini, Alessandra Grieco, Angelo Russo, Rosa Lucci, Giorgio Torella, Franco Berrino, Salvatore Panico, Carlo Vigorito

**Affiliations:** Division of Internal Medicine and Cardiac Rehabilitation, Department of Translational Medical Sciences, University of Naples “Federico II”, Via S. Pansini 5, 80131 Naples, NA Italy; Dipartimento di Medicina Clinica e Chirurgia, Federico II University, Naples, Italy; Medical Statistics Unit, Second University of Naples, Naples, Italy; Department of Preventive and Predictive Medicine, National Cancer Institute, Milan, Italy

**Keywords:** Cancer, Breast cancer, Exercise training, Autonomic function, Heart rate recovery, Cardiopulmonary exercise testing

## Abstract

**Purpose:**

To determine whether exercise training improves autonomic function in women with breast cancer (BC).

**Methods:**

Fifty-one patients (aged between 39 and 72 years) with a history of primary invasive BC within the previous 5 years and enrolled in the Mediterranean diet-based DIANA (Diet and Androgens)-5 Trial were subdivided in two groups: a ET group (n = 25) followed a formal ET program of moderate intensity (3 session/week on a bicycle at 60–70% VO_2peak_ for 3 months, followed by one session/week until 1-year follow-up), while a control group (n = 26) did not perform any formal ET. At baseline and after 1-year, all patients underwent cardiopulmonary exercise stress test (CPET). Heart rate recovery (HRR) was calculated as the difference between heart rate at peak exercise and heart rate at first minute of the cool-down period.

**Results:**

There were no significant differences between groups in baseline anthropometrical, BC characteristics, metabolic profile, CPET parameters and HRR. Compared to controls, at 1-year follow-up ET group showed a significant improvement in VO_2peak_ (from 12.6 ± 3.0 to 14.5 ± 3.3 ml/kg/min, p < 0.001; p < 0.001 between groups); and in HRR (from 17.6 ± 6.4 to 23.0 ± 8.3 beats/min, p < 0.001; p < 0.001 between groups). In ET group the changes in HRR directly correlated with changes in VO_2peak_ (r = 0.58, p = 0.002).

**Conclusions:**

Moderate intensity exercise training in BC survivors is associated with improvement of autonomic function. Whether the improvement of sympatho-vagal balance may favorably modulate some of the pathophysiological mechanisms implied in cancer evolution need further investigation.

## Background

Breast cancer (BC) is the most common cancer and cause of death in women worldwide. Diet and physical activity play a pivotal role on occurrence of BC (http://www.ncbi.nlm.nih.gov/pubmed/?term=diet+and+breast+cancer+and+meta-analysis; Ferrari et al. 2013; Buckland et al. 2013). Several studies have investigated and quite consistently reported a possible effect of physical activity on the incidence of primary BC (http://www.ncbi.nlm.nih.gov/pubmed/?term=physical+activity+and+breast+cancer+and+meta-analysis; Ibrahim and Al Homaidh 2011; Ballard-Barbash et al. 2012; McTiernan 2008); and secondary prevention studies after BC diagnosis consistently showed that the level of physical activity of BC women was correlated to an improvement in several cancer-related outcomes. In addition, exercise intervention studies have also suggested that a formal program of exercise training is also able to improve several cancer-related outcomes in BC survivors (Spence et al. 2010; Gleeson et al. 2011; Goh et al. 2012).

Previous studies showed that autonomic nervous system imbalance is associated with mortality in patients with cardiovascular disease (CVD) (Freeman et al. 2006). The heart rate response to, and recovery from, a bout of exercise is mediated by the dynamic interaction between the sympathetic and parasympathetic components of the autonomic nervous system (Freeman et al. 2006). Specifically, greater sympathetic tone predominates as heart rate increases during exercise, and vagal reactivation mediates the rate at which heart rate recovers after exercise (Freeman et al. 2006). Notably, the rate at which heart rate recovers from exercise (heart rate recovery, HRR) is associated with all-cause and cardiovascular mortality (Cole et al. 1999, 2000; Nishime et al. 2000).

There is evidence of a sustained increase in sympathetic activity and a reduction in parasympathetic input to the sino-atrial node in patients treated for early stage BC (Jones et al. 2012). Several studies have also shown that heart rate variability and baroreflex sensitivity is reduced among women with a history breast cancer (Meinardi et al. 2001).

This study tested the hypothesis that exercise training improves autonomic function as evaluated by HRR in BC women belonging to the DIANA (Diet and Androgens)-5 cohort.

## Methods

### Study population

The DIANA (Diet and Androgens)-5 study is a multicentre randomized controlled trial of the effectiveness of a diet based on Mediterranean and macrobiotic recipes and principles, associated with moderate physical activity, in reducing additional breast cancer (BC) events in women with early stage invasive BC at high risk of recurrence because of metabolic or endocrine milieu (Villarini et al. 2012). The overall design of the study has been described elsewhere (Villarini et al. 2012). We analyzed monocentric data (University of Naples “Federico II”) consisting of 51 patients randomized into two groups: 25 patients (51.8 ± 7.7 years, training group) were assigned to a structured exercise training intervention (3 times/week for the first 3 months, and once/week for the following 9 months); whereas 26 patients (53.9 ± 8.5 years, control group) received only general recommendations to adhere to the life-style intervention suggestions of the DIANA protocol. Our institutional ethical committee approved the protocol. The purpose of the protocol was explained to the patients and written informed consent was obtained from each patient before inclusion.

### Study protocol

At the study enrollment and after 12 months, all patients underwent anthropometrical and biochemical assessment, cardiovascular clinical examination, and cardiopulmonary exercise stress testing. Before randomization, all women received a leaflet illustrating the WCRF/AICR recommendations for cancer prevention (http://www.dietandcancerreport.org/cancer_prevention_recommendations/index.php). Training group patients were administered both the dietary and exercise training program as detailed below. After randomization, only the intervention group (training group, 25 patients) underwent, in addition to exercise training sessions, a counseling dietary program supported by cooking classes, reinforcing meetings and print materials. The counseling program aimed at increasing leisure-time physical activity level, controlling weight, and promoting a healthy and low calorie diet. Control group (26 patients) received only general recommendations on life-style.

### Anthropometrical and biochemical assessment

Body mass index (BMI) was used as a general measure of obesity and was calculated at the final visit as weight (kg) divided by height (m^2^). Waist circumference (WC), an index of abdominal obesity, was measured midway between the bottom of the rib cage and the top of the iliac crest. Blood pressure was measured twice after 5 min rest using a mercury sphygmomanometer. Anthropometric measurements were made with the subjects in indoor clothing and without shoes. Blood specimens were collected after 12- to 14-h fast, from 08:00 to 09:30 h, to reduce the influence of circadian variation. Total cholesterol, triglyceride and high-density lipoprotein (HDL) cholesterol levels were measured using standard enzymatic methods (Gentile et al. 2013). Low-density lipoprotein (LDL) cholesterol was calculated according to the Friedewald formula. Fasting glucose levels were enzymatically determined by the peroxidase method. Fasting insulin levels were determined by enzyme immunoassay (Ultrasensitive Insulin Elisa, Mercodia, Sweden). The homeostatic assessment model (HOMA) index was used to estimate insulin resistance and calculated as fasting serum insulin (mU/l) × fasting serum glucose (mM)/22.5, as previously described (Matthews et al. 1985).

### Cardiopulmonary exercise training testing

Respiratory gas exchange measurements were obtained breath by breath with the use of a computerized metabolic cart (Vmax 29C, Sensormedics, Yorba Linda, CA, USA) as detailed elsewhere (Giallauria et al. 2006a, b). Briefly, after a 1-min warm-up period at 0 W workload, a ramp protocol of 20 W/min was started and continued until exhaustion. The pedaling was kept constant at 55–65 revolutions/min. A 12-lead electrocardiogram (ECG) was monitored continuously during the test, and cuff blood pressure was manually recorded every 2 min. VO_2peak_ was recorded as the mean value of VO_2_ during the last 20 s of the test and was expressed in milliliters per kilogram per minute. Predicted VO_2peak_ was determined by use of a sex-, age-, height- and weight-adjusted and protocol-specific formula as previously detailed (Giallauria et al. 2006a, b). The ventilatory anaerobic threshold (VAT) was detected by use of the V-slope method. The VE versus VCO_2_ relationship was measured by plotting ventilation (VE) against carbon dioxide production (VCO_2_) obtained every 10 s of exercise (VE/VCO_2slope_): both VE and VCO_2_ were measured in liters per minute. The VE/VCO_2slope_ was calculated as a linear regression function, excluding the non-linear part of the relationship after the onset of acidotic drive to ventilation (Giallauria et al. 2006a, b). Heart rate (HR) and blood pressure (BP) at baseline and peak exercise, and heart rate during the cooling down phase after exercise were recorded. HRR was calculated as the difference between HR at peak exercise and heart rate at first minute of the cool-down period (Giallauria et al. 2008a, b).

### Exercise training program and cooking session

Training group patients were enrolled in an in-hospital structured exercise training program. The frequency goals of in-hospital exercise training sessions was three times/week for the first 3 months, and once/week for the following 9 months. Each session consisted of cycle or treadmill 30 min exercise, preceded by 5 min of warming and followed by 5 min of cooling down, at a work load of 75% of baseline peak VO_2_. This structured exercise training was on top of the general leisure-time physical activity recommendations or prescription of the general DIANA study (questionnaires, caloric control, etc., see above). From the 4th month to the 12th month, in-hospital exercise training sessions were reduced to one session/week, integrated with general leisure-time physical activity sessions, according to recommendations and prescription of the general DIANA study (Villarini et al. 2012). During cooking sessions, patients were invited cooking and eating some dishes prepared according to the WCRF/AICR recommendations and inspired by a macrobiotic Mediterranean diet (http://www.dietandcancerreport.org/cancer_prevention_recommendations/index.php).

### Statistical methods

Continuous variables were reported as either mean and standard deviation (SD) or median and range according to their distribution. The normality of residuals was tested with the Shapiro–Wilks test, and if residuals were not normally distributed the difference between HRR values was tested using the Wilcoxon test. Categorical variables were reported as percentage. Differences in characteristics of patients among groups were tested by means of one-way ANOVA and Pearson Chi square test for continuous and categorical variables, respectively. Data were analyzed using SAS version 9.2 (SAS Inc, Cary, NC, USA). All statistical tests were two-sided and p-values <0.05 were considered significant.

## Results

Baseline characteristics of the study population are shown in Table 1. There were no significant differences between groups in baseline anthropometrical, BC characteristics and metabolic profile (Table 1).

**Table 1:** Anthropometrical and clinical characteristics of the study population

	Training Group (n = 25)	Control Group (n = 26)	*P* value
Anthropometrics			
Age (years)	51.8 ± 7.7	53.9 ± 8.5	0.55
Waist circumference (cm)	94 ± 15	94 ± 12	0.66
Body mass index (kg/m^2^)	27.3 ± 5.2	28.2 ± 4.8	0.20
Breast cancer characteristics			
Presence of invasive carcinoma (%)	25/25 (100)	26/26 (100)	–
Node positivity (%)	48 (n = 12/25)	54 (n = 14/26)	0.58
Estrogen positivity (%)	80 (n = 20/25)	85 (n = 22/26)	0.78
Metabolic and hormonal profile			
Glycemia (mg/dl)	93.5 ± 12.5	92.2 ± 15.5	0.67
Insulin (ng/ml)	7.8 ± 4.0	8.2 ± 2.2	0.29
HOMA index	1.739 ± 1.403	1.925 ± 0.908	0.54
Total cholesterol (mg/dl)	205 ± 34	203 ± 32	0.85
LDL-cholesterol (mg/dl)	126 ± 35	123 ± 29	0.24
HDL-cholesterol (mg/dl)	54.3 ± 11.6	54.8 ± 12.7	0.63
Triglycerides (mg/dl)	100 ± 43	109 ± 52	0.13
Testosterone (nmol/l)	0.36 ± 0.36	0.38 ± 0.26	0.79

*HOMA* homeostatic assessment model, *LDL* low-density lipoprotein, *HDL* high-density lipoprotein.

The study period was similar in both groups. In training group, the average exercise intensity was 70 ± 2% of baseline VO_2peak_. No adverse events took place during any of training sessions in BC patients undergoing exercise training sessions.

Both groups were overweight at baseline (Table 1). At 1-year follow-up, a significant decrease in BMI (from 27.3 ± 5.2 to 26.6 ± 4.9 kg/m^2^, p = 0.02), in WC (from 94 ± 15 to 90 ± 13 cm, p = 0.005), and in systolic blood pressure (from 121 ± 17 to 115 ± 12 mmHg, p = 0.011), while no changes were observed among controls (Table 2). Baseline plasma lipids, glucose and insulin levels were normal and did not change at 1 year in both groups.

**Table 2:** Anthropometrics, metabolic and hormonal, cardiopulmonary and autonomic parameters

	Trained group (n = 25)		*P*-value	Control group (n = 26)		*P*-value	Differences in changes between groups
	Baseline	1-year		Baseline	1-year		
Anthropometrics							
Waist circumference (cm)	94 ± 15	90 ± 13	0.005	94 ± 12	95 ± 12	0.88	<0.001
Body mass index (kg/m^2^)	27.3 ± 5.2	26.6 ± 4.9	0.02	28.2 ± 4.8	28.3 ± 5.4	0.67	<0.001
Metabolic and hormonal profile							
Glycemia (mg/dl)	93.5 ± 12.5	92.8 ± 13	0.61	92.2 ± 15.5	90.6 ± 18.1	0.63	0.44
Insulin (ng/ml)	7.8 ± 4.0	8.0 ± 5.0	0.74	8.2 ± 2.2	7.9 ± 3.1	0.71	0.88
HOMA index	1.739 ± 1.403	1.822 ± 0.629	0.51	1.925 ± 0.908	1.825 ± 0.477	0.61	0.77
Total cholesterol (mg/dl)	205 ± 34	200 ± 29	0.66	203 ± 32	210 ± 26	0.42	0.62
LDL-cholesterol (mg/dl)	126 ± 35	122 ± 30	0.36	123 ± 29	126 ± 35	0.22	0.29
HDL-cholesterol (mg/dl)	54.3 ± 11.6	55.5 ± 9.7	0.78	54.8 ± 12.7	50.1 ± 8.7	0.19	0.52
Triglycerides (mg/dl)	100 ± 43	108 ± 42	0.53	109 ± 52	119 ± 46	0.60	0.41
Testosterone (nmol/l)	0.36 ± 0.36	0.40 ± 0.16	0.21	0.38 ± 0.26	0.33 ± 0.19	0.49	0.42
Cardiopulmonary parameters							
VO_2peak_ (ml/kg/min)	12.6 ± 3.0	14.5 ± 3.3	<0.001	12.8 ± 2.5	12.6 ± 2.8	0.55	<0.001
O_2_-pulse (ml/kg/min/beat-1)	6.2 ± 1.2	6.9 ± 1.2	<0.001	6.0 ± 1.4	6.2 ± 1.2	0.39	<0.001
VE/VCO_2slope_	29.7 ± 4.4	30.9 ± 3.9	0.12	30.1 ± 3.8	31.3 ± 4.9	0.46	0.44
Autonomic function							
Heart rate recovery	17.6 ± 6.4	23.0 ± 8.3	<0.001	16.4 ± 7.7	17.4 ± 7.8	0.35	<0.001

*HOMA* homeostatic assessment model, *LDL* low-density lipoprotein, *HDL* high-density lipoprotein, *VE/VCO*_*2slope*_ the minute ventilation-carbon dioxide production, *VO*_*2peak*_ peak oxygen consumption.

No differences in baseline cardiopulmonary parameters were found (Table 2). At baseline VO_2peak_ and O_2_-pulse were in the lower range (Weber class C) in both groups. In ET group VO_2peak_ (from 12.6 ± 3.0 to 14.5 ± 3.3 ml/kg/min, p < 0.001) and O_2_-pulse (from 6.2 ± 1.2 to 6.9 ± 1.2 ml/kg/min/beat-1, p < 0.001) improved at 1-year follow-up, respectively. No significant changes in VO_2peak_ (12.8 ± 2.5 to 12.6 ± 2.8 ml/kg/min, p = 0.55; p < 0.001 between groups), and in O_2_-pulse (from 6.0 ± 1.4 to 6.2 ± 1.2 ml/kg/min/beat-1, p = 0.39; p < 0.001 between groups) were observed at 1-year follow-up among controls.

No significant differences in HRR were found among groups (Table 2). ET group showed a significant improvement of HRR (from 17.6 ± 6.4 to 23.0 ± 8.3 beats/min, p < 0.001); whereas no significant changes were observed among controls (from 16.4 ± 7.7 to 17.4 ± 7.8 beats/min, p = 0.35; p < 0.001 between groups).

At 1-year follow-up, the level of physical fitness achieved in ET group as expressed by VO_2peak_ was inversely correlated with the 1-year value of BMI (r = −0.426, p = 0.001) and WC (r = −0.47, p < 0.001). In ET group the changes in HRR directly correlated with changes in VO_2peak_ (r = 0.58, p = 0.002).

## Discussion

The main finding of this study is that structured exercise training improves autonomic function in women with early stage invasive BC at high risk of recurrence because of metabolic or endocrine milieu.

Mounting evidences suggest that long-term autonomic imbalance is associated with increased risk of CVD and mortality in non-cancer populations (Cole et al. 1999, 2000; Nishime et al. 2000). There is evidence of a sustained increase in sympathetic activity and a reduction in parasympathetic input to the sino-atrial node in patients treated for early stage BC. The resting HR of early stage BC patients following the completion of primary adjuvant therapy is, on average, 9–16% higher compared to age-matched controls (Jones et al. 2012). Several studies have also shown that heart variability and baroreflex sensitivity is reduced among women with a history breast cancer (Meinardi et al. 2001). Accordingly, based on a growing understanding of bi-directional interactions between the sympathetic and parasympathetic efferent pathways, autonomic imbalance is one potential pathway involved in both the etiology and the clinical course of breast cancer therapy-induced CVD.

Despite the potential long-term consequences of autonomic dysfunction in women with BC, the development of safe and effective therapeutic strategies remains still elusive. Exercise training is one non-pharmacological therapy that may improve cardiovascular risk profile in the early BC setting; however, the mechanisms by which ET mitigates autonomic dysfunction are not fully understood. There is some clue that the central pathways responsible for decreasing sympathetic outflow and increasing cardiac vagal tone after ET are, in part, dependent on changes in the renin–angiotensin–aldosterone system (RAAS), nitric oxide (NO), and reactive oxygen species (ROS) (Scott et al. 2014). The signaling pathway RAAS plays a pivotal role in chemotherapy induced cardiotoxicity (Cadeddu et al. 2010). Since angiotensin II exerts powerful inhibitory effects upon the cardiac vagus nerve, its suppression or a precursor via ET could ultimately play an important role in the prevention of cardiac dysfunction. Breast cancer therapies also inhibit vascular NO release, consequently promoting vasoconstriction, increased peripheral resistance, and increased blood pressure (Facemire et al. 2009). Thus the up-regulation of NO with ET could improve autonomic function. Interestingly, there is evidence that ET improves peripheral arterial endothelial function (a surrogate measure of NO bioavailability) in women with operable BC receiving neoadjuvant doxorubicin (Jones et al. 2013). Finally, chemotherapy-induced generation of reactive oxygen species (ROS) is the central mediator of several adverse acute and chronic biological effects in the cardiovascular system, including alterations in autonomic outflow (Minotti et al. 2004). Accordingly, attenuation of ROS generation and/or activity holds considerable therapeutic promise. A recent study found that ET during chronic anthracyclines exposure in mice reduced serum and cardiac levels of ROS and attenuated LV remodeling suggesting a protective role of ET of cardiac cells against chemotherapy-induced toxicity through ROS inhibition (Dolinsky et al. 2013).

The nervous system plays a significant role in control of cancer growth and metastasis (Ondicova and Mravec 2010). Stress causes alterations in the immune system, the neuroendocrine system and sensory nerve functions, which in turn could contribute to cancer mortality (Erin et al. 2004a, b, 2006; Ercan et al. 2006; Spiegel et al. 1998). Stressful life events can accelerate the progression of existing cancer (Chida et al. 2008). How cancer-induced systemic changes, as well as stress of the disease, alter neuroimmune functions is not fully understood. Interestingly, the adrenal gland, due to its centrality on the hypothalamus, pituitary, and adrenal gland neuroendocrine axis, plays a crucial role in regulation of the stress response by providing a link between the neuroendocrine and immune systems. There is evidence that adrenal response to certain cytokines is suppressed in patients with cancer, which may alter appropriate immune response. The vagus nerve and capsaicin-sensitive sensory neurons seem to play a crucial role in the regulation of adrenal functions, as well as the immune system. Stimulation of the vagus nerve increases the activity of nerve fibers innervating the adrenal medulla (Niijima 1992). Inactivation of capsaicin-sensitive sensory nerve fibers increases post-stress plasma cortisone levels (Ulrich-Lai et al. 2003). In addition, epinephrine released from the adrenal medulla increases the activity of afferent vagal fibers, and these fibers may in turn influence the activity of sympathetic nerves modulating the release of epinephrine, as well as the stress response (Mravec et al. 2006). The vagus nerve also directly regulates the immune system, such that activation of the vagus nerve inhibits the inflammatory response (Zracey 2002) and alters (CD4)+ T cell functions (Karimi et al. 2010). In an elegant study, Erin et al. (2013) showed that vagotomy enhances adrenal metastasis. Interestingly, the layer most affected by the tumor burden was the medulla, thus resulting in sympathetic deregulation, and consequently immune dysfunction, and in turn further enhancing the metastatic ability of the carcinoma. Therefore, the vagus nerve plays a significant role in defense against cancer by inhibiting cancer metastasis. Since there is strong evidence that lifestyle modification (diet and physical activity) have positive impact on cancer outcomes (Mishra et al. 2012), the improvement of sympatho-vagal balance might be at the basis of these beneficial prognostic effects.

Mounting evidences showed that despite normal cardiac function, BC women have significant and marked impairments in cardiopulmonary function (Jones et al. 2012; Sweeney et al. 2006). Notably, we found that baseline VO_2peak_ and O_2_-pulse values were in the lower range (Weber class C) in both cohorts; however, BC women undergoing ET significantly improved their cardiopulmonary functional capacity. There is solid evidence that the level of physical exercise is associated to improved outcome in BC patients, included cancer related and total mortality (Ballard-Barbash et al. 2012). Therefore, the present study adds to the growing literature for exercise training as a valid, safe and additive therapeutic strategy in cancer patients.

Some limitations of the present study should be considered. The present study was not designed at evaluating cancer outcomes (i.e. mortality, recurrences). In addition, training patients underwent moderate intensity exercise program: whether an exercise program of higher intensity might have additional clinical and prognostic value should be clarified. Despite the aforementioned limitations, this study has several unique strengths. This is the first study enrolling high-risk BC survivors (women with early stage invasive BC at high risk of recurrence because of metabolic or endocrine milieu) to 1-year exercise intervention showing significant improvement of autonomic function.

## Conclusions

In conclusion, moderate intensity exercise training in BC survivors improves autonomic and cardiopulmonary function. Whether the improvement of sympatho-vagal balance may favorably modulate some of the pathophysiological mechanisms implied in cancer evolution need further investigation.
